# Considering neurophysiological mechanisms of dual-tasking in people with multiple sclerosis: an exploratory, cross-sectional small-N study

**DOI:** 10.3389/fneur.2026.1648874

**Published:** 2026-01-30

**Authors:** Kristen E. Plandowski, Sarah J. Donkers, Zahra Moslemi, Maruf Ahmad, Cameron S. Mang

**Affiliations:** 1 Faculty of Kinesiology and Health Studies, University of Regina, Regina, SK, Canada; 2 School of Rehabilitation Sciences, College of Medicine, University of Saskatchewan, Saskatoon, SK, Canada

**Keywords:** cognitive-motor interference, cortical silent period, corticospinal inhibition, neurodegenerative disease, neurorehabilitation, transcranial magnetic stimulation

## Abstract

**Introduction:**

Dual-tasking is an emerging topic of study within the field of multiple sclerosis (MS) rehabilitation. Past research on dual-task performance among people with MS (PwMS) is limited by methodological differences and minimal consideration of underlying neurophysiology. Related studies suggest that changes in inhibitory neural activity in the motor cortex may support dual-task performance in healthy adults, as assessed using transcranial magnetic stimulation (TMS). Other TMS work indicates that MS alters corticospinal inhibition, but how and whether it is modulated during dual-tasking in PwMS is unknown. The objective of this exploratory, cross-sectional small-N study was to explore whether changes in corticospinal inhibition that occur during dual-tasking may be different in PwMS compared to non-MS controls.

**Methods:**

Six PwMS (4F; 45.17 ± 15.74 years) and three non-MS controls (2F; 42.33 ± 16.62 years) performed motor and cognitive tasks under single- and dual-task conditions. Each dual-task included a core motor task, which involved maintaining a steady pinch grip force. Performance of this core motor task allowed for assessment of corticospinal inhibition during task performance via measurement of the cortical silent period elicited by TMS. Tasks combined with the core motor task included holding a string of numbers and/or number letter combinations in working memory and a toe-tapping task. Several versions of the tasks were presented alongside the core motor task, each providing different levels of novelty and complexity. Dual-task performance was measured as dual-task cost, considering task performance and cortical silent period duration. Analyses included descriptive statistics and, in line with a small-N study design, examination of individual data.

**Results:**

There was no evidence of greater cognitive-motor interference in PwMS relative to non-MS controls. Task novelty and complexity effects between PwMS and non-MS controls were similar. Despite behavioral similarities, PwMS displayed greater changes in cortical silent period under dual-task conditions compared to non-MS controls that were accentuated under motor-motor dual-task conditions.

**Discussion:**

Findings suggest that while PwMS and non-MS controls may perform similarly during dual-tasking, the neurophysiological mechanisms involved may be different. Further work is needed to elucidate the impact of MS-related changes in the corticospinal system on dual-tasking.

## 1 Introduction

Multiple sclerosis (MS) is a chronic, neurodegenerative disease of the central nervous system (CNS) characterized by demyelination, neuroinflammation, and focal lesions in both the brain and spinal cord (1). Many people living with MS (PwMS) experience both motor (e.g., paresis) and cognitive (e.g., sensory processing deficits) impairments that affect their daily lives. Prior research has indicated that PwMS demonstrate challenges with cognitive-motor interference (2) and indicates that many PwMS experience difficulties performing two tasks at once (3), also known as dual-tasking. Most research regarding dual-tasking in PwMS has focused on assessing performance-based outcomes and relationships between cognitive-motor function related to walking and balance (4, 5). However, given that MS is a disease that affects the CNS, there is a need to also understand what CNS regions and neural circuitry contribute to dual-tasking and how they might be affected by MS.

Transcranial magnetic stimulation (TMS) is a technique commonly used to study the excitability of the motor cortex and the corticospinal tract (6, 7) and has been employed as a tool to investigate how neural activity may be altered under dual-tasking situations (8, 9). TMS can be used to assess numerous aspects of the motor system, including how the CNS balances inhibition and excitation to generate appropriate motor control (10). Prior studies using dual-task paradigms in healthy adults have demonstrated that the concurrent performance of two or more tasks results in changes to corticospinal inhibition (8) and task performance (11) compared to a single-task. In these studies, corticospinal inhibition was measured by using TMS to elicit the cortical silent period (CSP), a transient reduction in electromyographic (EMG) activity in an active muscle. The duration of the CSP is considered to reflect the activity of gamma-aminobutyric acid (GABA_B_) receptors within the motor cortex and spinal cord (12, 13). A systematic review and meta-analysis reported alterations in CSP duration with dual-tasking in healthy populations, suggesting that dual-task performance may be partly dependent on changes in corticospinal inhibition that regulate motor control (8). Evidence presented in this review (10, 14, 15) and a later study (9) indicated that corticospinal inhibition was increased when TMS was applied during continuous performance of two tasks.

Prior research has indicated that inflammation present in MS affects synaptic functioning linked to TMS outcomes (16) and that imbalances between excitatory and inhibitory transmission in the CNS (17) are correlated to disability levels (18). Furthermore, evidence of alterations in cortical functioning as measured through TMS suggests abnormalities even in the early stages of the disease (6). Dual-task paradigms used to examine cognitive-motor interference in PwMS have demonstrated deteriorations in task performance, such as decreases in motor and/or cognitive function (2, 19–21) with some conflicting evidence as to whether such performance losses are amplified in PwMS (22). While it has been postulated that both cognitive and motor function in MS may be negatively impacted by abnormal cortical and corticospinal excitability, no studies to our knowledge have specifically examined how or whether corticospinal activity is altered during dual-tasking in this population.

The primary purpose of this preliminary study was to explore changes in corticospinal inhibition and performance during dual-tasking and cognitive-motor interference in PwMS and non-MS control participants. Secondary purposes, guided by a framework for dual task taxonomy developed by McIsaac and colleagues (23), were to determine whether differing task novelty and complexity levels would impact the magnitude of these changes measured via dual-task costs, and to consider interference effects during a dual motor-motor task. We hypothesized that PwMS would demonstrate greater dual-task costs and more pronounced changes in corticospinal inhibition during dual-tasking than non-MS controls. Secondarily, we expected that differences between PwMS and non-MS controls would be more apparent under dual-task conditions with high task novelty and complexity. We expected to observe similar effects with the dual motor-motor task.

## 2 Materials and methods

### 2.1 Study overview and design

**Figure 1:**
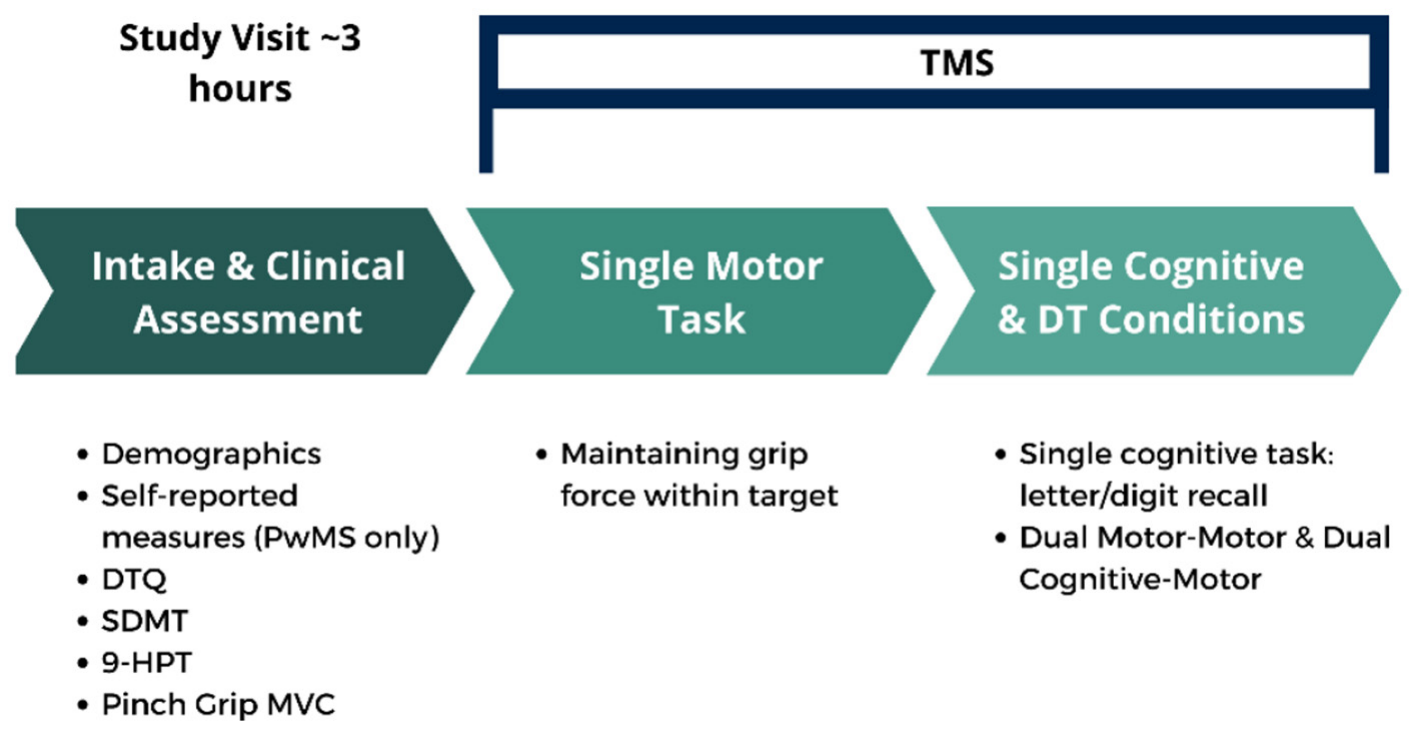
Study overview. DT, dual-task; TMS, transcranial magnetic stimulation; DTQ, dual-task questionnaire; SDMT, symbol digit modalities test; 9-HPT, 9-hole peg test; MVC, maximum voluntary contraction.

All participants provided their informed consent to participate in the study (University of Regina Research Ethics Review Board, File #: 2021-171). The study used a cross-sectional design to investigate dual-tasking in PwMS and age-matched, control participants without MS. All participants were initially screened for eligibility over the phone. Eligible and consenting participants then attended a 3-h data collection session at the University of Regina. During this visit, participants completed intake forms and a clinical assessment. A series of laboratory-based motor and cognitive tasks were then performed under single and dual-task conditions. Briefly, single-tasks included a pinch grip force motor task, a verbal recall cognitive task, and a rhythmic toe-tapping motor task. Tasks were combined to assess dual-task cost during cognitive-motor and motor-motor task performance. TMS was delivered during performance of the pinch grip motor task under single and varied dual-task conditions to determine potential task-dependent alterations in corticospinal inhibition. An overview of the study design is depicted in Figure 1.

### 2.2 Participants

Adults reporting physician-diagnosed MS of any type, and age- and sex-matched volunteers without MS were recruited to the study. To be eligible to participate, it was necessary for PwMS to demonstrate the ability to perform the motor tasks used in the study (i.e., pinch grip and toe-tapping). Exclusion criteria for PwMS were: relapse within the last 6 months or no reported difficulty with dual-tasking in everyday life (i.e., self-report of ≤ 1 on the DTQ-Expanded) (24, 25). Additional exclusion criteria applicable to both PwMS and non-MS controls were: any contraindications to TMS or, receiving treatment with antiepileptic drugs or long-lasting benzodiazepines, due to the potential for influences on motor cortex inhibition (26). Finally, non-MS control participants were free of any central or peripheral neurological condition.

### 2.3 Procedures

#### 2.3.1 Clinical assessment

All participants completed a study intake form to gather information on demographics (e.g., age, sex), clinical characteristics (e.g., type of MS, disease duration), and medications. A series of questionnaires and assessments were then conducted. First, an expanded version of the original 10-item, self-report Dual-Task Questionnaire (DTQ) (27) with five additional questions (28) was administered to characterize dual-tasking ability in the everyday lives of participants. Next, the Symbol Digit Modalities Test (SDMT) was used to assess information processing speed in participants. The oral version of the test was conducted using standardized instructions. The total score was calculated using the number of correctly coded responses given by participants in 90 s (29). Participants with MS also completed three additional questionnaires, which included the Patient-Determined Disease Steps (PDDS) (30), the SymptoMScreen (31), and the Multiple Sclerosis Impact Scale (MSIS-29) (32).

To assess fine motor function of the upper limbs, participants performed the 9-hole Peg Test (9-HPT). Both dominant and non-dominant hands were tested twice using pre-established instructions explained to participants by the researcher. A 9-HPT Asymmetry score (%) between the dominant and non-dominant hands was calculated (33). Lastly, pinch grip strength was measured by a custom force transducer in which participants were instructed to pinch as forcefully as they could for approximately 5 s using their self-reported dominant hand. Two trials were completed with ~60 s between each trial, and the highest force (N) achieved was considered the maximal voluntary contraction (MVC). If maximal force outputs differed considerably (>25%), a third trial was conducted.

#### 2.3.2 TMS assessment

When applying TMS, a Magstim 200^2^ stimulator (Magstim, Carmarthenshire, UK) was used to deliver monophasic pulses over the primary motor cortex with a figure-of-eight coil (Magstim D70 Alpha Coil) targeting activation of the first dorsal interosseous (FDI) muscle of the self-reported dominant hand. All participants with MS self-reported that their dominant hand had not changed after their MS diagnosis, and these reports aligned with 9-HPT performance in which all participants demonstrated better performance with their self-reported dominant, compared to non-dominant, hand. Brainsight Neuronavigation software was used to guide coil position and orientation, and responses elicited in the FDI were measured using surface EMG. Brainsight Neuronavigation utilizes a position sensor to track both the participant and the coil, projecting a visualization of their position in relation to each other on a screen and ensuring that the same target area is stimulated throughout the TMS session. EMG was recorded with 1 cm × 1 cm surface recording electrodes placed in a belly-tendon montage. The skin was prepared for electrode placement by wiping the area with 70% isopropyl alcohol pads. Once the area had dried, electrodes were placed over the belly of the FDI muscle, the ulnar styloid process, and the proximal interphalangeal joint of the index finger. EMG data was collected using LabChart software (LabChart 8.0; AD Instruments, Colorado Springs, CO), with signals sampled at 4,000 Hz, pre-amplified (1,000 × ) and band-pass filtered at 10–1,000 Hz using a Powerlab data acquisition system and two bioamplifiers (AD Instruments). Data was recorded in a 550 ms sweep from 100 ms before to 450 ms after delivery of each TMS pulse.

Prior to beginning the motor and cognitive tasks, baseline TMS variables were collected. Using standard procedures (34), the “hotspot,” resting motor threshold (RMT), and active motor threshold (AMT) for the target FDI muscle was determined. To identify the “hotspot,” suprathreshold pulses were delivered over the primary motor cortex contralateral to the FDI, in which the coil placement that produced the largest response was sampled to ensure constant orientation throughout the study. The RMT was determined by applying single pulses at a subthreshold intensity that did not elicit a motor evoked potential (MEP). Intensity was increased in 1%−5% increments until MEPs consistently produced peak-to-peak amplitudes of 50 μV or higher. The intensity was then reduced in 1% increments until an intensity was reached where 5/10 pulses produced an MEP of 50 μV or higher and identified as the RMT. Once the RMT was determined, “hotspot” procedures were reiterated to ensure that the location of TMS application was optimal. With this confirmed hotspot, AMT was determined as follows. Participants received visual feedback of pinch grip force and were guided to maintain a contraction with their self-reported dominant hand of 15% of their MVC. The methods used to determine the RMT were also used to determine the AMT but with a threshold MEP amplitude of 200 μV. The AMT was used as a reference value for calculation of stimulation intensity used to elicit the CSP. Note that the pinch grip force task involved in the TMS assessment was concurrently used as the core motor task for dual tasks described in subsequent sections.

Next, participants completed a series of motor and cognitive tasks (described in detail below). TMS was delivered during performance of all tasks that included the core motor task of maintaining a steady pinch grip force of 15% MVC with the self-reported dominant hand. For this task, participants were provided with continuous visual feedback of a target force “bar” and a force-controlled cursor presented on a computer screen. The mid-line of the target force bar was set at 15% of the pinch grip MVC force, with upper and lower bounds of ±5. Delivering TMS during the pinch grip allowed quantification of CSP duration concurrent with task performance. For these measurements, a total of 20 single pulses of TMS were delivered at an intensity of 155% of the AMT with an interstimulus interval of 8–10 s. Suprathreshold stimulation at an intensity of 155% of the AMT was chosen for CSP measurement. Past work has demonstrated that higher stimulation intensities elicit longer CSP durations (35). We used AMT as the reference for determining CSP intensity in anticipation of potential challenges in eliciting MEPs at rest in PwMS due to reduced cortical excitability. Although the use of RMT is less common for determining CSP trial intensities, it is an accepted approach (34, 36) that has been used in recent studies of PwMS (18, 37).

**Figure 2:**
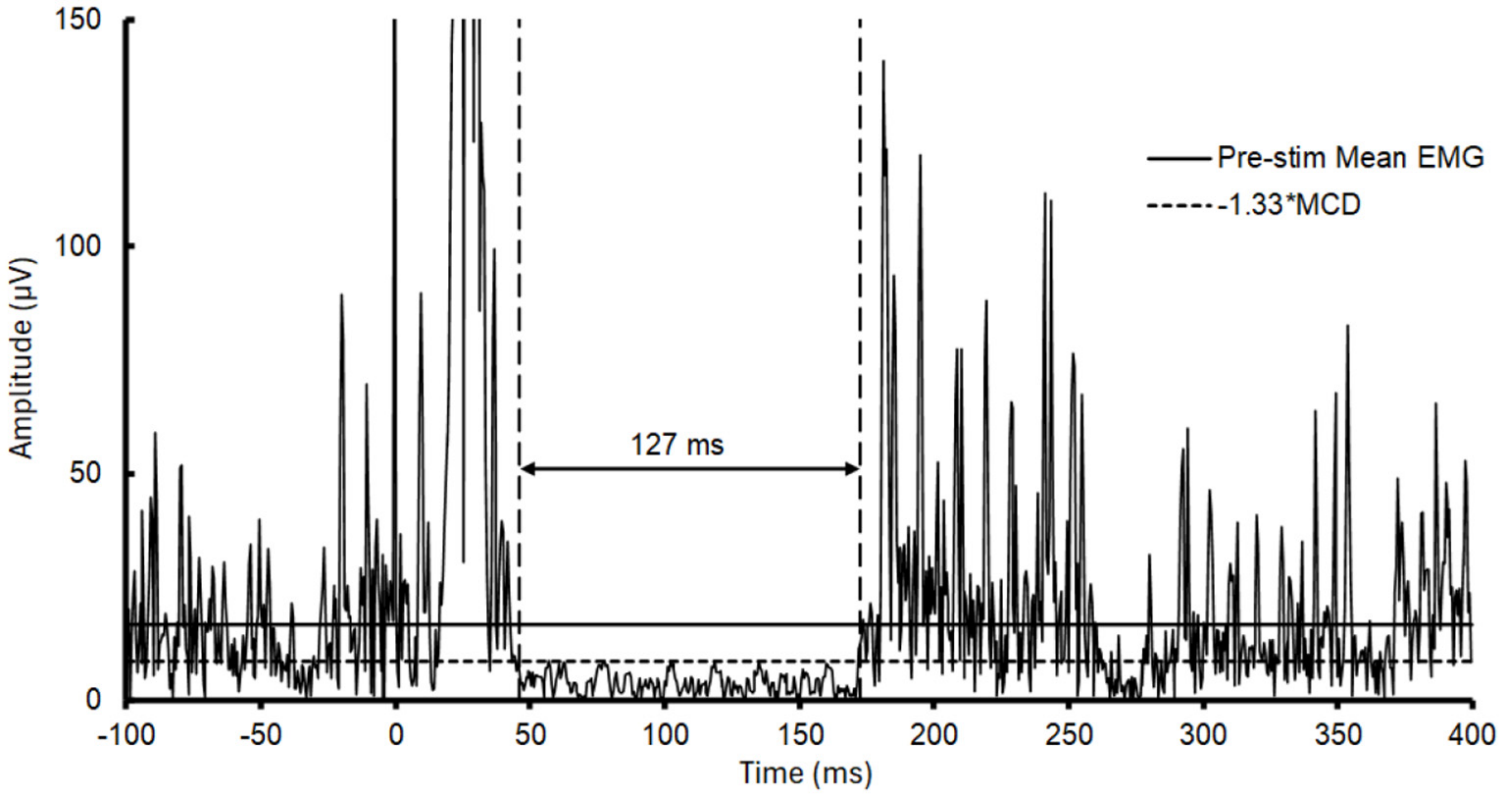
Measurement of the cortical silent period (CSP) duration. The panel shows rectified electromyography (EMG) for the first dorsal interosseous (FDI) muscle for a representative trial in which the CSP was identified and the duration measured from a participant with multiple sclerosis (MS). The transcranial magnetic stimulation (TMS) pulse was delivered at 0 ms, which is followed by a motor evoked potential (MEP). The lower variation limit used for CSP identification was calculated as mean pre-stimulus EMG – (|MCD| × 1.33).

A custom MATLAB script (Mathworks, Natick, MA) was used to calculate the duration of the transient reduction in muscle activity (i.e., the CSP) that followed the elicited MEP in each trial. Our approach was guided by the objective, graphical procedures described by Garvey et al. (38) and Hupfeld et al. (36). Using the script, EMG was downsampled from 4,000 to 2,000 samples per second, rectified, and upper and lower variation limits were calculated by determining the mean consecutive difference (MCD) of EMG data points occurring every 0.5 ms for the 100 ms prior to stimulation: mean pre-stimulus EMG ± (|MCD| × 1.33). These limits spanned 87% of possible pre-stimulus EMG data points (equivalent to 1.5 standard deviations). Onset and offset of the CSP were then determined with the following criteria: (1) onset was the first of five consecutive data points (2.5 ms) to fall below the lower variation limit; (2) offset was the first data point to reach above the lower variation limit if at least 50% of data points in the following 5 ms window were also above the limit (38). The time between the onset and offset was quantified as the CSP duration for each trial (Figure 2) and averaged for each participant under each experimental condition. Briefly, Garvey et al. (38) referred to the use of mean pre-stimulus EMG ± (|MCD| × 2.66) as variation limits; however, in this past work the CSP was collected while participants maintained a maximal muscle contraction rather than the 15% MVC contraction used here and in other studies of PwMS (18, 37, 39) and dual-tasking (9). In analyzing our data, we determined that with the level of muscle contraction used, limits more restrictive than 1.33^*^MCD reached a “floor” that precluded identification of visible silent periods. Additionally, Garvey et al. (38) and Hupfeld et al. (36) suggested that CSPs be identified from an averaged EMG trace, whereas other recommendations indicate that CSPs may be identified from either an averaged EMG trace or by averaging trial-by-trial measurements (34, 40). With a 15% MVC baseline muscle contraction, we found that amplitude cancelation attenuated pre-stimulus mean EMG when considering an averaged EMG trace, and thus we performed trial-by-trial measurements.

For a CSP to be identified, it was necessary for an MEP to be evoked and followed by a measurable reduction in EMG activity that could be identified by our objective procedures. In some instances, a CSP could not be identified, resulting in the trial being discarded from analyses. On average, three trials were discarded within each set of 20 stimulations (range: 0–12; median: 2). Online monitoring of pinch grip force and communication between experimenters ensured that all stimuli were delivered when pinch grip force was within the specified target range.

#### 2.3.3 Motor and cognitive tasks

All participants completed a series of motor and cognitive tasks comprised of three main tasks (two motor, one cognitive). A total of 13 tasks were performed consisting of three core tasks and 10 combinations and/or variations. Each of these tasks were performed alone and in combination with another task. Combinations included dual cognitive-motor tasks, and, secondarily, dual motor-motor tasks. Task variations were designed to alter task novelty and complexity as shown in Table 1.

**Table 1:** Task variations and combinations.

**Type of tasks**	**Task novelty**	**Task complexity**	
		**Low**	**High**
Single motor		Pinch grip^*^	
Single cognitive	Low	Memorizing string of five numbers	Memorizing string of 10 numbers
	High	Memorizing string of five number/letter combinations	Memorizing string of 10 number/letter combinations
Dual cognitive-motor	Low	Maintaining a steady pinch grip force while memorizing string of five numbers	Maintaining a steady pinch grip force while memorizing string of 10 numbers
	High	Maintaining a steady pinch grip force while memorizing string of five number/letter combinations	Maintaining a steady pinch grip force while memorizing string of 10 number/letter combinations
Dual motor-motor	Low	Maintaining a steady pinch grip force while tapping toe to a self-selected rhythm	Maintaining a steady pinch grip force while steadily tapping toe as quickly as possible
	High	Maintaining a steady pinch grip force while tapping toe to an external rhythm with a consistent tempo (1 beat/s)	Maintaining a steady pinch grip force while tapping toe to an external rhythm with varying tempo

^*^Task novelty and complexity were not altered in this condition.

The core motor task (i.e., the pinch grip force task) was performed without any variations throughout the duration of the trial and was completed as the first task by all participants. As noted above, for this task participants were instructed to perform a pinch grip with the self-reported dominant hand and maintain a steady force level using visual feedback of a target force “bar” presented on a computer screen for the duration of the TMS data collection. The mid-line of the target force bar was set at 15% of the pinch grip MVC force, with upper and lower bounds of ±5%. Participants performed the pinch grip task for the time needed to collect the aforementioned TMS data (i.e., 20 stimuli with 8–10 s inter-stimulus interval = ~180–200 s). Rest breaks of a maximum of 15 s were provided if participants were unable to maintain the target force level for the duration of the TMS collection. This pinch grip task was performed first as a single-task, and subsequently in combination with both a cognitive task (dual cognitive-motor), another motor task (dual motor-motor), and several variations of each.

The cognitive task required participants to review a string of numbers and/or number/letter combinations presented on a piece of paper over 30 s. Participants then were instructed to hold the string in their mind for a period of time, and then verbally recite as much of the string as possible. Four variations of this task that manipulated novelty and complexity through string number/letter composition and length, respectively, were completed, as described in Table 1. The low novelty condition strings only consisted of number combinations, while the high novelty condition strings contained both number and letter combinations. Low complexity strings were five numbers/letters in length, while the high complexity strings were 10 numbers/letters in length. The tasks were completed separately as single cognitive tasks, and in combination with the pinch grip force motor task (dual cognitive-motor task). The time period during which participants “held” the string in their working memory and maintained the pinch grip force concurrently was ~200 s based on the time required to collect the TMS data. This time was consistent across single and dual-task conditions.

Creation of the cognitive task for the current work was based on past research indicating that dual-task paradigms involving working memory tasks led to high levels of cognitive-motor interference among both PwMS and controls (41). Working memory has been identified as a cognitive domain that can become overloaded during dual-tasking (42), and therefore may produce more noticeable effects under experimental conditions than other cognitive tasks. Likewise, the task was designed to be similar to the “Digit Span Working Memory Test,” which is known to have good to excellent reliability under dual-task conditions (43); yet, it was necessary to create a modified version of this task to allow for study of potential novelty and complexity effects (23) and for delivery over an extended period of time, which supported TMS assessment. Consequently, the task was unique to this study and is not supported by validation or reliability evidence, which limits generalizability and necessitates cautious interpretation of findings.

The second motor task was performed in combination with the core motor task to form the dual motor-motor task. Given the length of the study protocol (i.e., 3 h) and the inclusion of the dual motor-motor task as an extension from the primary purpose (i.e., examining corticospinal inhibition during cognitive-motor interference), this task was not performed separately as a single task. This approach is similar to other dual-tasking research that focuses on changes in core task performance only (9), and importantly, core motor task and CSP data was still available for analyses under both single- and dual motor-motor task conditions. For this motor task, participants were required to perform a toe-tapping task on a pedal force transducer using the foot ipsilateral to the pinch grip task. Four variations of the task that manipulated novelty and complexity through toe-tapping with self-selected or external rhythms and varied pace, respectively, were performed (see Table 1). Duration of toe-tapping was again tied to the time required to collect the TMS data and was ~200 s per condition. Up to two rest breaks of a maximum of 15 s were provided on request of the participant. Low novelty conditions required participants to tap their toe to a self-selected beat (tapping as quickly as possible at a steady pace), while the high novelty conditions required participants to tap their toe to an external beat played from a speaker (a steady external beat at a tempo of 1 beat per second and an external beat varying in tempo). The external beat utilized in the high novelty conditions was a standardized track overlaid into a LabChart (LabChart 8.0; AD Instruments, Colorado Springs, CO) template that was used for all participants.

**Figure 3:**
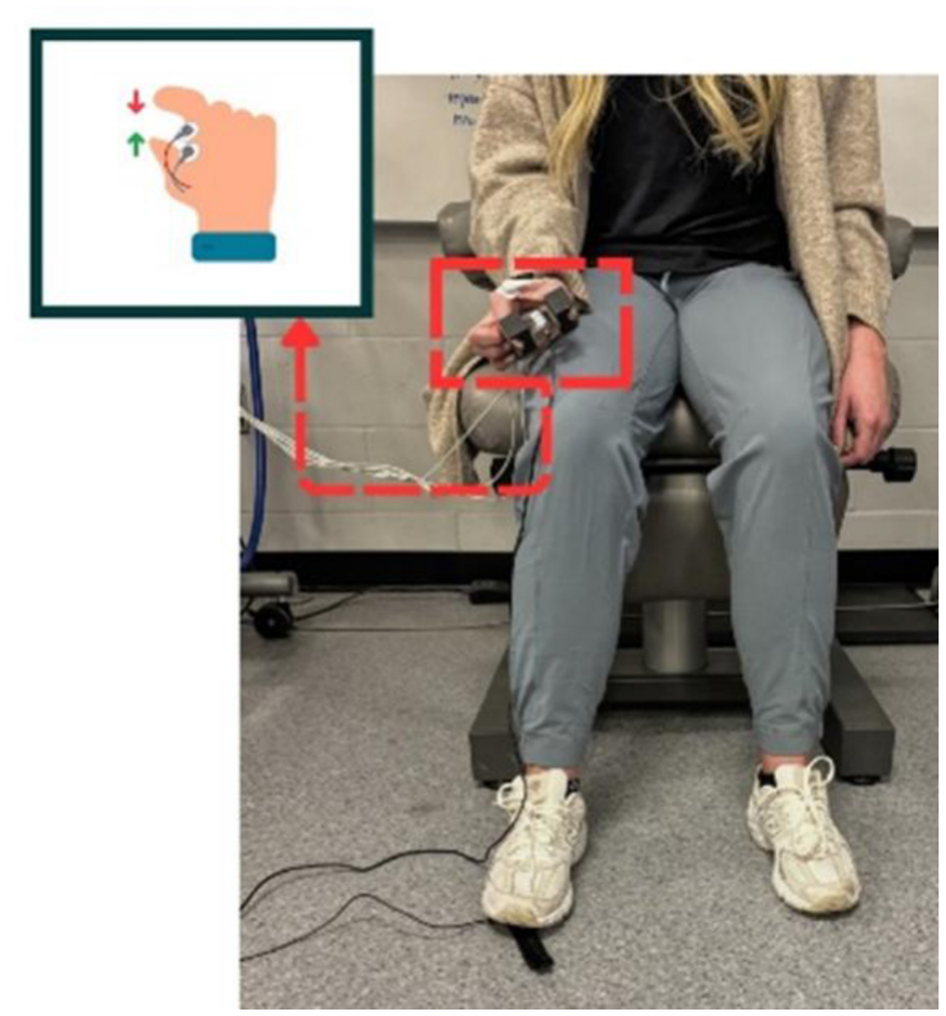
Participant set-up with pinch grip force transducer, electrode placement over the target muscle, and the pedal force transducer tapped by participants.

While all sessions began with the core motor task (i.e., the pinch grip task), the order of performance of the remaining tasks, their combinations, and the variations were determined based on a randomized schedule for each participant. As described in the *TMS Assessment*, single pulses of TMS were delivered during all tasks that included the core pinch grip motor task. In order to maintain consistency of the protocol delivery, pre-defined instructions for each task were used by the researcher. For dual-tasks, participants were instructed to perform both tasks to the best of their ability and not prioritize one over the other. The participant setup is shown in Figure 3.

Task performance was determined for each of the motor and cognitive tasks. Performance of the core motor task was evaluated by determining the proportion of time maintained within the target force bar during the ~200 s trial. Core cognitive task performance was measured as the number of correct items recalled from the string of number and/or number/letter combinations immediately following the end of the trial. Performance measurement in the secondary motor task was dependent on the task novelty and complexity version. In the low novelty-low complexity version (i.e., tapping at self-selected rhythm), performance was calculated as the variability (i.e., standard deviation) in time between toe-taps, where a greater value indicates a less consistent or more variable performance. The low novelty-high complexity variation (i.e., steady toe-tapping as quickly as possible) was measured using the same method. In both the high novelty-low complexity (i.e., tapping to consistent external rhythm) and high novelty-high complexity (i.e., tapping to varied external rhythm) versions, performance was measured as variability (i.e., standard deviation) in response time based on the difference in time to toe-tap relative to the auditory beat. Tapping to the consistent external rhythm was measured manually through LabChart (LabChart 8.0; AD Instruments, Colorado Springs, CO), while tapping to the varying external rhythm was measured using a custom MATLAB (Mathworks, Natick, MA) script written in-house. As with the pinch grip task, performance was measured across the entire ~200 s trial.

#### 2.3.4 Dual-task costs

Task specific interference for cognitive-motor and motor-motor dual-tasks and each version were calculated (see Table 2), where a greater absolute value indicates a larger change in performance. The same general equations were applied to calculate a dual-task cost related to the change in CSP duration determined through the TMS assessment; however, the equation was amended such that a negative dual-task effect reflected a longer CSP under dual-task compared to single-task conditions. As such, a negative value represents a decline in performance or lengthening of the CSP during dual-task relative to single-task conditions (i.e., interference or dual-task cost), while a positive value represents an improvement in performance or shortening of the CSP during dual-task relative to single-task conditions (i.e., dual-task facilitation) (44).

**Table 2:** Equations used to determine task performance and dual-task cost.

**Single-task performance**		
Core motor task	$\frac{Grip\;force\;time\;maintained\;within\;target}{Total\;time\;of\;task\;performance}\times 100\%$	
Core cognitive task	$\frac{\#\;of\;correct\;responses}{Total\;length\;of\;string}\times 100\%$	
**Dual-task cost**		
Motor DT effect	$\frac{Motor\;DT-Motor\;ST}{Motor\;ST}\times 100\%$	(–) Inhibition (+) Facilitation
Cognitive DT effect	$\frac{Cognitive\;DT-Cognitive\;ST}{Cognitive\;ST}\times 100\%$	(–) Inhibition (+) Facilitation

ST, single-task; DT, dual-task. Table adapted from Longhurst et al. (44). For dual-task cost calculations with the cortical silent period (CSP), the equation was amended such that a negative dual-task effect reflected a longer CSP under dual-task compared to single-task conditions.

### 2.4 Statistical analyses

We conducted an *a priori* sample size calculation informed by previous studies that examined the effect of dual-tasking on corticospinal inhibition in healthy adults (9) and differences in TMS measures between PwMS and non-MS controls (45). With expectations of a large effect size (Cohen's *d* = 1.4), a desired β = 0.80, and α = 0.05, it was determined that a sample size of *n* = 20 (10 PwMS and 10 non-MS controls) would be sufficient to detect differences in the effect of dual-tasking on corticospinal inhibition between PwMS and non-MS controls. With this sample size, we intended to conduct a series of two-way (Task Condition × Group) mixed analyses of variance for the dual cognitive-motor and dual motor-motor tasks, with dual-task cost of measures of interest as dependent variables. Challenges with participant recruitment precluded efforts to attain the planned sample size and data collection was ultimately completed in six PwMS and three non-MS controls. Recruitment challenges included: pandemic restriction and post-pandemic discomfort with in-person research participation, willingness to undergo TMS assessment, and the length of the data collection session (~3 h; see Section 4.5). Study and analysis approach was therein revised to reflect an exploratory, small-N study (46).

In applying a small-N study approach, the approach to statistical analyses was amended. Group descriptive statistics (mean ± standard deviation) for demographic and clinical assessments are provided (Table 3) to characterize the sample, but analysis of TMS assessments and task performance centered on individual values. Individual characteristics are also provided in Supplementary Table S1. When interpreting individual dual-task costs in terms of task performance, a 10% change in performance was interpreted as meaningful based on dual-task assessments that commonly use this 10% benchmark (e.g., Timed Up and Go Dual-task) to identify individual impairment in dual-tasking (47). Interpretation of dual-task costs in terms of corticospinal inhibition (i.e., CSP duration) considered a 20% intra-individual change as meaningful based on a report of the minimal detectable difference in various TMS measures, including the CSP (48). In this prior work, the minimal detectable difference in CSP was 28.9 ms for CSPs in the range of 134.70–145.14 ms (i.e., 19.9%−21.6% change) for older adults. In individuals with stroke, the minimal detectable difference was 49.74 ms for CSPs in the range of 181.75–181.87 ms (i.e., 27.3% change) elicited from the lesioned hemisphere and 30.55 ms for CSPs in the range of 169.66–170.96 ms (i.e., 17.8%−18.0% change) elicited from the non-lesioned hemisphere (48). The minimal detectable difference is a concept that is typically applied to identify “true” intra-individual change in a measure as a result of an intervention (49). Here, the concept is applied to allow categorization of any changes in behavioral or neurophysiological measures as a result of dual- rather than single-task conditions as meaningful based on typical clinical approaches (47) and related evidence describing test-retest reliability of CSP duration (48). Such detailed, intra-individual observation and comparison is often not feasible in the context of a larger sample study (46). Importantly, application of the minimal detectable difference in this manner represents an exploratory approach that was applied to support interpretation for the current small-N study. We do not recommend the use of 20% as a definitive threshold for identifying meaningful change in other cross-sectional CSP work.

**Table 3:** Participant characteristics.

**Parameter**	**PwMS (*n* = 6)**	**Controls (*n* = 3)**
Age (years)	45.17 ± 15.74	42.33 ± 16.62
Sex	4 F/2 M	2 F/1 M
Height (m)	1.75 ± 0.14	1.68 ± 0.07
Weight (kg)	90.47 ± 14.40	111.12 ± 23.03
MS phenotype	4 RRMS/2 PPMS	–
Disease duration (years)	6.33 ± 6.35	–
Taking DMTs, *n* (%)	5 (83.3%)	–
PDDS	4.00 ± 2.10	
SymptoMScreen	21.17 ± 7.11	–
MSIS-29	72.00 ± 26.08	–
DTQ-expanded	2.57 ± 0.70	2.44 ± 0.47

Data reported as mean ± SD. F, female; M, male; RRMS, relapse remitting multiple sclerosis; PPMS, primary progressive multiple sclerosis; DMT, disease modifying therapies; PDDS, patient-determined disease steps; MSIS-29, multiple sclerosis impact scale; DTQ-expanded, dual-task questionnaire-expanded. PDDS ranges from 0 to 8, with a higher score indicating greater perceived disability. The SymptoMScreen is scored out of 72, with higher scores indicating greater impairment. MSIS-29 is scored out of 100, with higher scores indicating greater levels of disability. DTQ-Expanded is scored on a Likert scale (range 0.0–4.0) and averaged across 15 questions, with a higher score indicating greater perceived difficulties with dual-tasks.

## 3 Results

### 3.1 Participant characteristics

Six PwMS and three non-MS control participants matched for age and sex participated in the study. Participant characteristics are provided in Table 3. Briefly, PwMS included four females and two males with an average disease duration of 6.33 (±6.35, range: 1–8) years. PwMS were diagnosed with relapsing-remitting MS (RRMS, *n* = 4) and primary progressive MS (PPMS, *n* = 2), and all but one were taking pharmacological MS disease modifying therapies (DMTs; *n* = 5). All participants were ambulatory and self-reported right-sided hand and foot dominance. In Table 4, mean clinical and TMS assessment values are provided. Considering all participants, PwMS and non-MS controls scored similarly on the SDMT, but PwMS demonstrated slower 9-HPT performance with the non-dominant hand and generally lower pinch grip maximum voluntary contraction force. Regarding baseline and single-task TMS measures, PwMS had both a higher RMT and AMT. Although variable, CSP duration was shorter on average in PwMS (101.53 ms ± 90.80) than in non-MS controls (143.93 ms ± 23.99).

**Table 4:** Clinical and TMS assessment.

**Parameter**	**PwMS (*n* = 6)**	**Controls (*n* = 3)**
SDMT (# correct)	56.50 ± 18.25	56.33 ± 17.67
9-HPT-D (s)	21.13 ± 3.84	19.98 ± 5.02
9-HPT-ND (s)	36.06 ± 17.42	20.50 ± 6.49
9-HPT-assymetry (%)	−66.64 ± 64.45	−1.60 ± 7.34
MVC (N)	74.1 ± 20.4	107.6 ± 12.8
RMT (%MSO)	60.00 ± 9.34	48.00 ± 9.00
AMT (%MSO)	55.67 ± 11.36	44.33 ± 10.60
CSP duration (ms)	101.53 ± 90.80	143.93 ± 23.99

Data reported as mean±SD. SDMT, symbol digit modalities test; 9-HPT-D, 9-hole peg test dominant hand; 9-HPT-ND, 9-hole peg test non-dominant hand; MVC, maximal voluntary contraction; RMT, resting motor threshold; AMT, active motor threshold; MSO, maximum stimulator output; CSP, cortical silent period. Mean CSP values reported from values obtained during single motor task (i.e., pinch grip force) performance.

### 3.2 Dual-task costs

#### 3.2.1 Dual cognitive-motor

**Figure 4:**
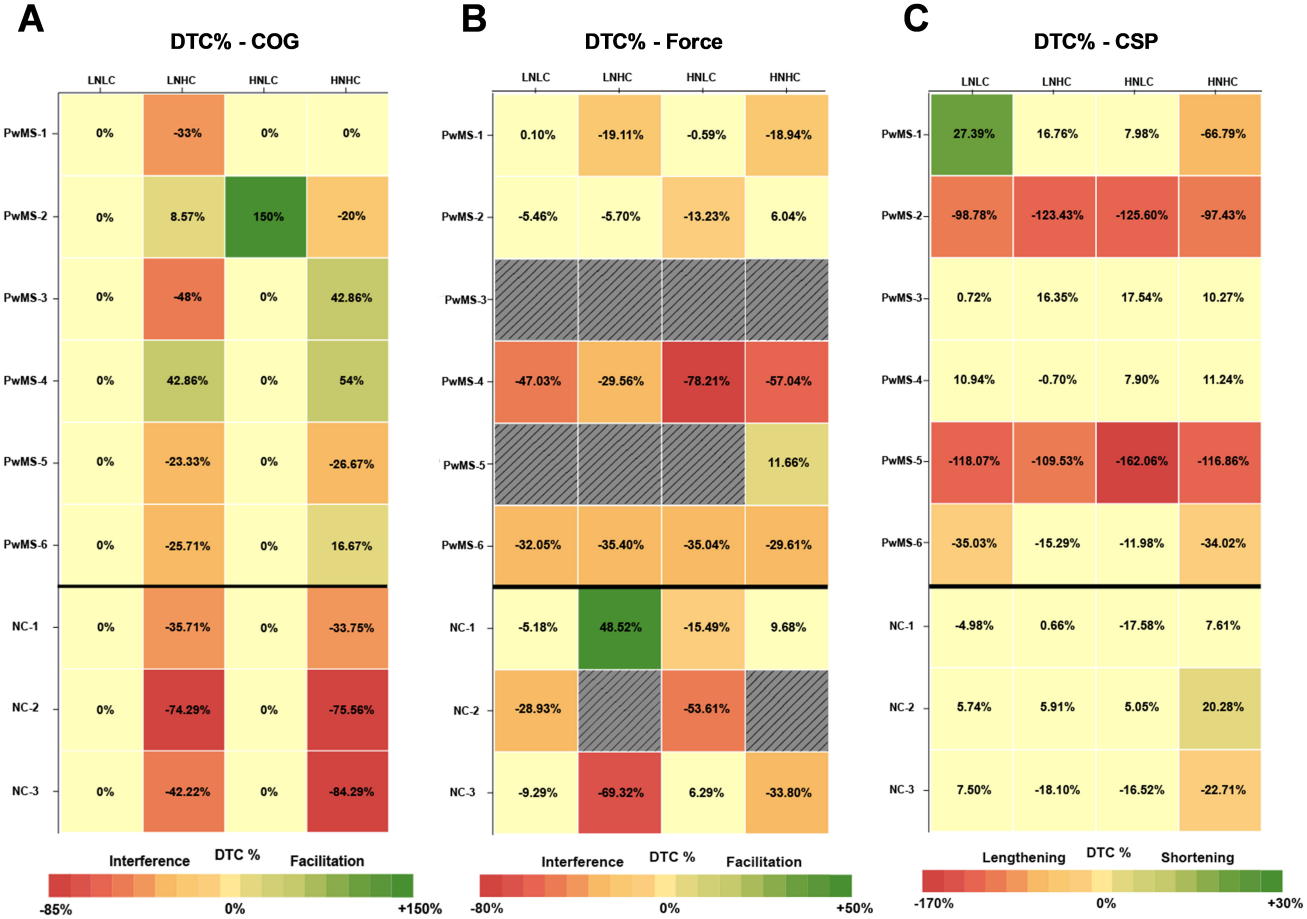
Dual-task cost % (DTC%) of cognitive task performance **(A)**, pinch grip force task performance **(B)**, and cortical silent period (CSP) duration **(C)** during the dual cognitive-motor (DCM) task conditions. Positive values (green) denote improved performance (facilitation) and shortened CSPs in the dual-task condition relative to single-task performance. Negative values (orange-red) denote poorer performance (interference) and lengthened CSPs in the dual-task condition relative to single-task performance. “0%” values (yellow) indicate that there was no change between single- and dual-task conditions. The shading of colors represents the magnitude of change in dual-task cost (%) and is visually depicted in the legend. Each gradient change represents a change of 10% for the cognitive and pinch grip force tasks and a change of 20% in CSP duration (see Section 2.4), with increasingly red shades representing greater interference or CSP lengthening. The solid black line differentiates between the participants with MS (PwMS) and the non-MS controls (NC). Gray regions represent missing or incomplete data. Due to experimenter error, force grip data was unreadable for PwMS-3, for PwMS-5 during DCM-LNLC, LNHC, and HNLC conditions, and for NC-2 during the DCM-LNHC and -HNHC conditions, accounting for 25% of grip force data collected during the DCM task. DTC %, dual-task cost %; COG, cognitive; CSP, cortical silent period; DCM, dual cognitive-motor; LNLC, low novelty/low complexity; LNHC, low novelty/high complexity; HNLC, high novelty/low complexity; HNHC, high novelty/low complexity; PwMS, person with MS; NC, non-MS control.

The dual-task cost (%) of cognitive recall performance during the dual cognitive-motor task conditions can be viewed in Figure 4A. PwMS and non-MS controls generally did not display performance changes in either of the low complexity conditions, which both involved recall of shorter strings of number/letter combinations. PwMS appeared to experience a lesser interference effect and in some cases a facilitation effect in the high complexity dual cognitive-motor conditions while non-MS controls experienced interference to a greater extent. In Figure 4B, the dual-task cost of the pinch grip force task performance during the dual cognitive-motor conditions is presented. There were few clear trends in dual-task cost of pinch grip force performance, besides a generally negative dual-task cost. Across conditions, PwMS demonstrated an average dual-task cost for pinch grip force task performance of −22.89% ± 23.80, and non-MS controls displayed a dual-task cost of −15.11% ± 33.69. When considering the CSP measurements (Panel C), four of six PwMS displayed at least one condition with a clear dual-task cost in CSP duration. Two PwMS (PwMS-2 and PwMS-5) demonstrated lengthening of CSP durations across all dual-task conditions. Changes in CSP duration with dual-tasking were less evident among non-MS controls. On average across conditions, PwMS demonstrated a dual-task cost in CSP duration of −41.19% ± 60.69, while non-MS controls displayed a dual-task cost of −2.26% ± 13.51. The novelty and complexity of the task conditions did not clearly impact the CSP dual-task costs.

**Figure 5:**
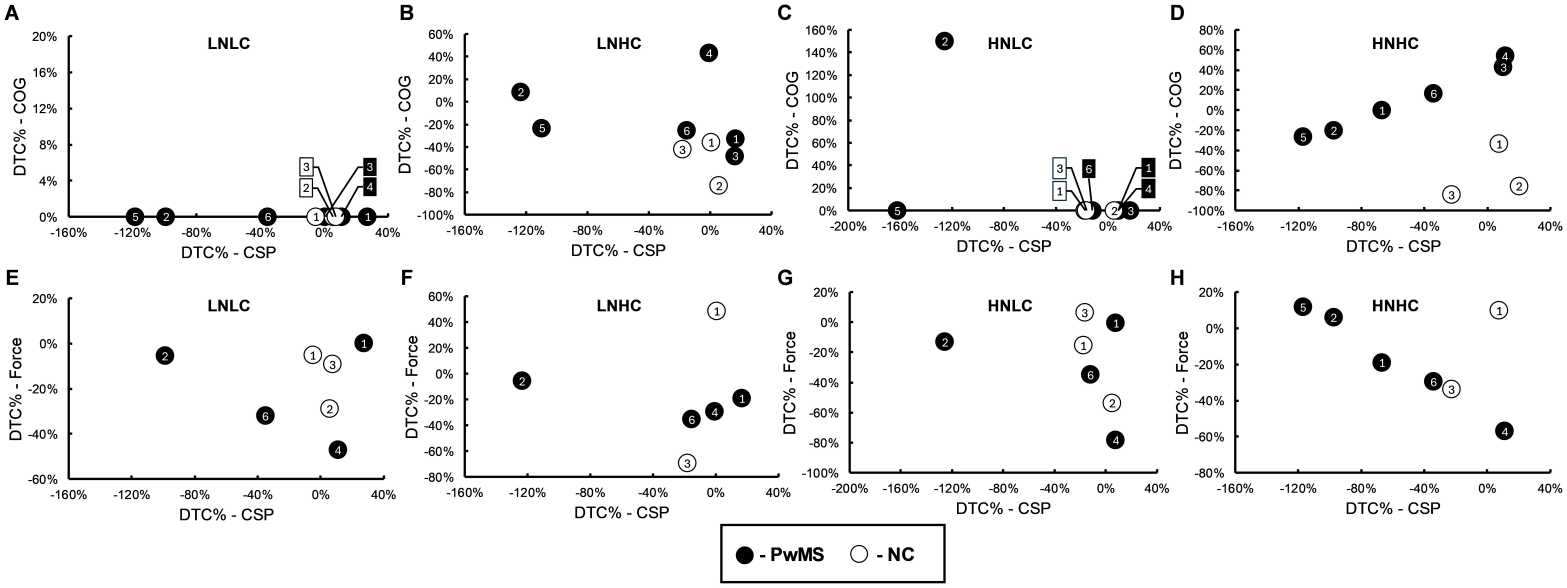
Scatterplots of dual-task cost % (DTC%) of CSP duration (*x*-axis) with DTC% of cognitive task performance [*y*-axis, **(A–D)**] and pinch grip force task performance [*y*-axis, **(E–H)**] during the dual cognitive-motor (DCM) task. Plots are presented for each level of cognitive task novelty and complexity. Data points are labeled according to each participant (i.e., filled marker labeled 1 corresponds to PwMS-1; open marker labeled 1 corresponds to NC-1). Rectangular labels with lines connecting to data points are used when markers overlap. DTC %, dual-task cost %; DCM, dual cognitive-motor; LNLC, low novelty/low complexity; LNHC, low novelty/high complexity; HNLC, high novelty/low complexity; HNHC, high novelty/low complexity; PwMS, participants with MS; NC, non-MS controls.

In Figure 5, relationships between dual-task costs and CSP modulation are provided. In panels D and H, data from the high novelty-high complexity task conditions are presented. These plots demonstrate that, under certain conditions, lengthening of the CSP may be related to interference in cognitive recall performance and facilitation of pinch grip force task performance in PwMS.

#### 3.2.2 Dual motor-motor

**Figure 6:**
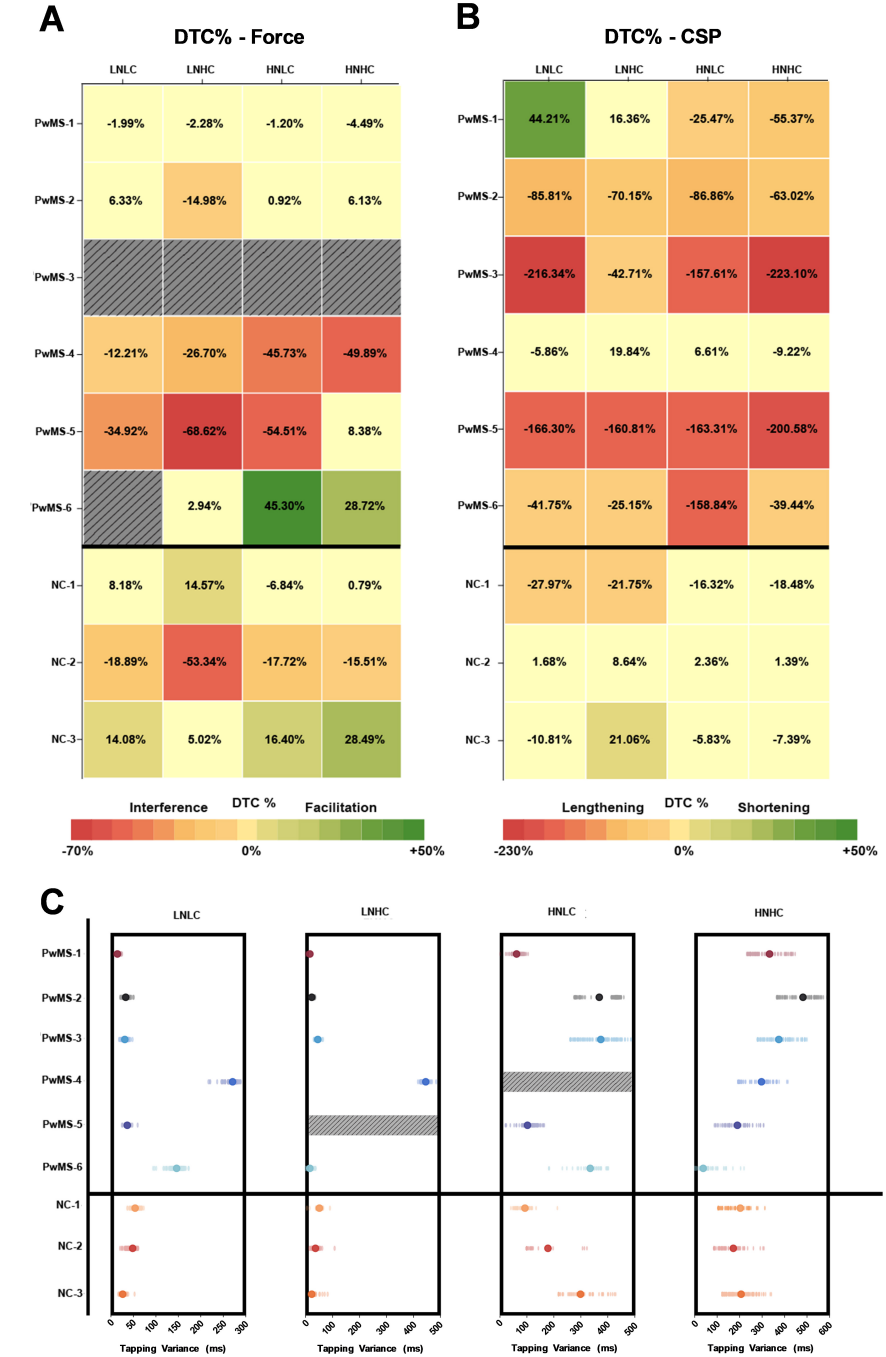
Dual-task cost % (DTC%) of pinch grip force task performance **(A)** and cortical silent period (CSP) duration **(B)** during the dual motor-motor (DMM) task conditions. Values, colors, and shading definitions are consistent with the Figure 4 caption. **(C)** Variance in toe-tapping performance during the DMM is presented, as toe-tapping under single task conditions was not collected and precludes presentation of DTC%. Solid dots (filled in circle) represent participant's individual variance in performance for each toe-tapping condition. The blurred circles around each solid dot represent the individual data points used to determine the variance. Due to experimenter error, force grip data was unreadable for PwMS-3, and for PwMS-6 during the DMM-LNLC condition, accounting for 14% of force grip data collected during the DMM task. Due to experimenter error, toe-taps were unreadable during PwMS-5′s DMM-LNHC condition and PwMS-4′s DMM-HNLC condition, accounting for 5.6% of the toe-tapping data. DTC %, dual-task cost %; CSP, cortical silent period; DMM, dual motor-motor; LNLC, low novelty/low complexity; LNHC, low novelty/high complexity; HNLC, high novelty/low complexity; HNHC, high novelty/low complexity; PwMS, person with MS; NC, non-MS control.

**Figure 7:**
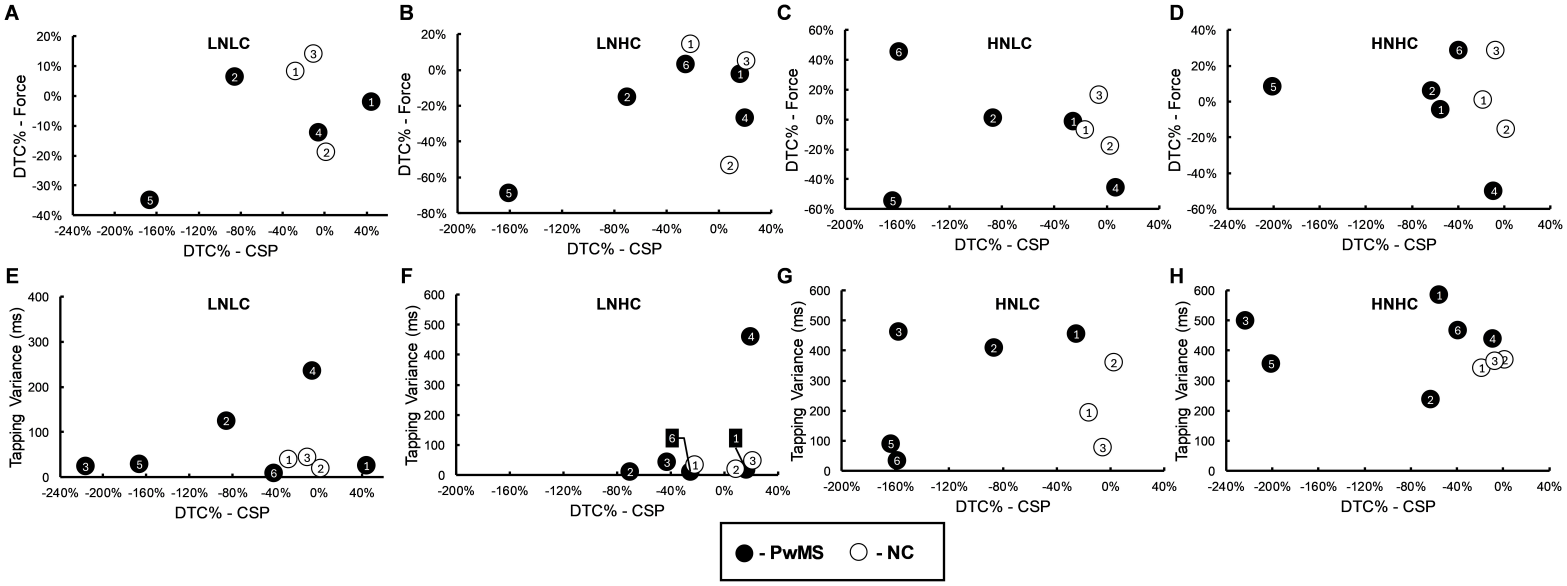
Scatterplots of dual-task cost % (DTC%) of CSP duration (*x*-axis) with DTC% of pinch grip force task performance [*y*-axis, **(A–D)**] and toe-tapping task variance [*y*-axis, **(E–H)**] during the dual motor-motor (DMM) task. Plots are presented for each level of toe-tapping task novelty and complexity. Data points are labeled according to each participant (i.e., filled marker labeled 1 corresponds to PwMS-1; open marker labeled 1 corresponds to NC-1). Rectangular labels with lines connecting to data points are used when markers overlap. DTC %, dual-task cost %; DMM, dual motor-motor; LNLC, low novelty/low complexity; LNHC, low novelty/high complexity; HNLC, high novelty/low complexity; HNHC, high novelty/low complexity; PwMS, person with MS; NC, non-MS control.

In Figure 6, the dual-task cost of the pinch grip force task performance (Figure 6A) and the CSP (Figure 6B) during the dual motor-motor conditions are depicted in the same manner as for the dual cognitive-motor tasks in Figure 4. As with the dual cognitive-motor task, there were again few clear trends in dual-task cost of pinch grip force performance. A generally negative dual-task cost was observed, besides in PwMS-6 and two non-MS controls, who showed evidence of facilitation effects. When averaging across all conditions, PwMS had a slightly greater dual-task cost in pinch grip force performance (−11.52% ± 28.97) than non-MS controls (−2.06% ± 22.03). Considering the CSP measurements, the majority of PwMS displayed a dual-task cost indicating change, and typically a lengthening, in CSP duration under dual-task conditions. In contrast, non-MS control participants generally did not demonstrate clear change in CSP durations under dual-task relative to single-task conditions. On average across all conditions, PwMS demonstrated a dual-task cost in CSP duration of −79.61% ± 81.03, while non-MS controls displayed a dual-task cost of −6.12% ± 13.97. The novelty and complexity of the condition did not impact the CSP dual-task cost in a clear pattern. Performance of the toe-tapping component to the dual motor-motor task is illustrated in Figure 6C. The toe-tapping tasks were performed only under dual-task conditions, so dual-task costs could not be calculated. For most participants, the high novelty conditions resulted in more variable performance as compared to the low novelty conditions, with a more notable impact on PwMS. Overall, trends in performance differences between PwMS and non-MS controls were not well-defined. In Figure 7, relationships among dual-task costs in pinch grip force task performance, toe-tapping variance, and CSP modulation are plotted, demonstrating no distinct associations.

## 4 Discussion

The purpose of this study was to conduct a preliminary exploration of changes in corticospinal inhibition and performance during dual-tasking among PwMS and non-MS controls. Secondarily, we considered whether dual-task costs were more apparent under dual-task conditions with differing levels of complexity and novelty. Given the exploratory, small-N nature of the present study, trends and patterns in the results were analyzed and inspected on an individual basis (46), revealing several preliminary observations. First, cognitive performance was not as negatively impacted by dual-task conditions as expected for PwMS. Average SDMT scores for the sample were within the normative range (29), but there was some potential indication of a “cognitive-first” prioritization strategy. Second, while no clear trends appeared in pinch grip force task performance, the majority of participants displayed some level of interference as demonstrated by generally negative dual-task costs. Finally, PwMS displayed increases in corticospinal inhibition as measured by the dual-task cost of CSP duration under dual-task conditions that were accentuated under the motor-motor dual-task conditions. Findings contradict our hypotheses predicting greater dual-task costs in performance among PwMS and effects of task novelty and complexity, but support postulation that corticospinal inhibition is altered and may be involved in dual-tasking in PwMS.

### 4.1 Exploratory, cross-sectional small-N design

When interpreting the findings of the current study, the small sample size is a critical consideration. Certainly, randomized controlled trials and prospective cohort studies with large samples are the most widely regarded study designs for generation of evidence in health research (50). Nevertheless, some aspects of these approaches also constrain their application. Perhaps most pertinent here, the emphasis on reporting average effects from group-level statistics in these gold-standard approaches may restrict the relevance of overall findings to specific individuals (51). In short, individual differences can be masked by a focus on group effects. An advantage of small-N designs is that they allow researchers to potentially identify factors and outcomes critical to individual participants (46). In the current work, repeated measurements were taken under multiple conditions (i.e., single- and varied dual-tasks) and compared within each participant. Such detailed, individual observation and comparison is often not feasible in the context of larger sample studies. Although generalizability of the findings from a single small-N study is limited, scientific contributions of this type of work include initiation of a series of direct replication small-N studies, integration into meta-analyses and other knowledge synthesis approaches, and hypothesis generation for future work (46).

A number of situations in which small-N studies are considered appropriate and useful in rehabilitation research have been identified, including: (i) low-prevalence conditions where it may not be feasible to recruit participants for sufficiently-powered between-groups analysis, (ii) conditions with the potential for substantial variability in the magnitude of responses, and (iii) unique circumstances in which the unique or preliminary nature of protocols precludes large-scale application (46). These situations apply to the current work, respectively, as follows: (i) MS is a relatively low-prevalence condition −36 people per 100,000 globally and, even in Canada, which has among the highest MS rates in the world, just 290 per 100,000 (52, 53); (ii) MS is widely known for its variability in clinical presentation (54); and, (iii) no prior work to our knowledge has used TMS to investigate the physiological basis of dual-tasking in PwMS.

Feasibility for recruitment for this study was challenged by the COVID-19 pandemic, when significant barriers to recruitment included public health restrictions, participants' levels of comfort in social spaces, and a lack of transportation options. In some cases, potential participants were also not eligible due to inability to perform the motor-motor dual-task, or lack of willingness to undergo TMS assessment. Despite the small sample size, our revised small-N approach examined individual data to provide in-depth exploration of dual-tasking study protocols and effects in this population.

### 4.2 Baseline cortical silent period duration

CSP duration was shorter on average among PwMS than non-MS controls within our sample. Early studies indicated that CSPs recorded from upper extremity muscles were prolonged in PwMS relative to non-MS controls (12, 55), and this finding was replicated recently (56). However, other studies have reported either no difference in CSP durations between PwMS and non-MS controls (45) or a shortened CSP among PwMS (57). Likewise, in one of the aforementioned studies, it was specified that 69% of PwMS presented with CSP abnormalities, and that while the majority of those abnormalities involved a lengthening of the CSP, a lack of CSP or a shortened CSP was also observed in some PwMS (12). Expanding on this observation, another recent study demonstrated that CSP changes in PwMS may depend on clinical phase (58). PwMS in the remitting phase presented with a lengthened CSP and those in the relapsing phase showed a shortened CSP relative to non-MS controls (58). Thus, CSP changes appear to be variable across PwMS and potentially dependent on individual factors, and in our small sample the observation of generally shorter CSPs compared to non-MS controls is not unparalleled. While differences in TMS procedures and CSP analysis methods pose difficulties for comparison across studies (36), the average CSP duration of 101.53 ms ± 90.90 reported here is within durations reported elsewhere for PwMS, which span group averages of approximately 40–170 ms (12, 18, 37, 39, 55, 56). Further, the mean CSP duration for non-MS controls found here (143.93 ms ± 23.99) appears long relative to some literature (55, 56) but is consistent with other work (48, 59).

### 4.3 Cognitive-motor dual-task

In this study, participants performed short-term recall cognitive tasks varying in novelty and complexity while simultaneously performing a motor task involving maintenance of a steady pinch grip force. In general, cognitive performance was unaffected amongst all participants during the low complexity conditions of the cognitive-motor dual-task, while conditions of higher complexity resulted in dual-task effects for both PwMS and non-MS controls. In contrast to our hypothesis, PwMS generally demonstrated less cognitive interference (i.e., a less negative dual-task cost) and, in some cases, even facilitation effects (i.e., a positive dual-task cost), whereas non-MS controls only experienced interference effects. These results contrast with the findings of Postigo-Alonso and colleagues (60), in which only PwMS displayed a decrease in cognitive performance during walking, while non-MS controls did not display any performance changes. Methodological differences may contribute to discrepancies in findings, specifically regarding the type of cognitive and motor tasks used and instructions regarding prioritization. For instance, the cognitive task used here had some overlapping demands with the SDMT (i.e., working memory), which many PwMS have been regularly exposed to through standardized clinical testing. Additionally, the pinch grip force motor task was performed with the self-reported dominant hand, which could be postulated to be relatively robust to negative dual-task effects due to automaticity of control. Further, in the study by Postigo-Alonso et al. (60), the added challenge and required postural control demands of a dynamic activity such as walking while dual-tasking may change the response (and how attention is allocated) due to factors such as environmental navigation and moving through space (61).

A conceptual framework proposed by Plummer and Eskes (61) may also provide insight into the current findings. Specifically, the authors suggested that task prioritization may be selected as a strategy in which factors such as risks and rewards are considered. Considering this framework, the study conducted by Postigo-Alonso et al. (60) in which one motor task consisted of walking as fast as possible can be contrasted with the current study in which participants performed motor tasks while seated. It could be argued that the paradigm used in this study presented less “risky” options when choosing how to allocate attentional resources during dual-tasking. Therefore, PwMS may have chosen to prioritize the cognitive task in the present study because both tasks were perceived as relatively low-risk. On the other hand, in a different dual-task walking study, Allali and colleagues (19) found that participants with RRMS demonstrated poorer motor task performance but maintained cognitive task performance, suggesting that cognitive prioritization may be evident even in walking dual-tasks in this population. Nevertheless, in this work only two participants with MS (PwMS-4 and PwMS-6) demonstrated notable losses in pinch grip force task performance during the cognitive-motor task, and therefore we cannot conclude that a clear prioritization of the cognitive task occurred.

Although trends in pinch grip force performance during the cognitive-motor dual-tasks were not well-defined across PwMS and non-MS controls or novelty and complexity conditions, we observed that most participants displayed at least a slight interference effect or negative dual-task cost. Observation of similar dual-task costs between PwMS and non-MS controls is consistent with findings of a systematic review by Learmonth et al. (22), which suggested that both PwMS and non-MS controls experience cognitive-motor interference and that related declines in performance do not differ greatly in magnitude between the two populations. Critically, the authors speculated that despite similar dual-tasking motor performance deficits, there may be neurophysiological differences that are overlooked in studies that focus solely on dual-task performance and functional assessments (22).

Our use of TMS in the current study supported investigation of possible neurophysiological distinctions. In contrast to the similarity in pinch grip force performance among PwMS and non-MS controls under dual-task cost conditions, apparent differences in dual-task costs of CSP duration (i.e., corticospinal inhibition) were observed between PwMS and non-MS controls. As shown in Figure 4, four of six PwMS experienced one or more dual cognitive-motor task conditions in which CSP duration was lengthened relative to the single-task measurement, with PwMS-2 and PwMS-5 demonstrating a lengthening of the CSP under all conditions. On the other hand, non-MS controls displayed minimal lengthening of CSP duration despite evidence of more negative cognitive dual-task costs compared to PwMS. In Figure 5, relationships observed under the high novelty-high complexity condition suggest that CSP lengthening under dual-task conditions may be associated with cognitive task interference and motor task facilitation. Although similar associations were not observed under other task conditions, the association between lengthened CSP duration and better motor performance is aligned with other work (10, 14, 62, 63). For example, in one study longer CSPs recorded from the tibialis anterior muscle were related to better turning performance in PwMS (63). Similar associations were observed between corticospinal inhibition and other measures of motor performance in older adults, whereas the inverse relationship was reported in younger adults (10, 14, 62, 64). Although preliminary, our current work adds the finding of a concomitant association between CSP lengthening and cognitive interference. In any case, our findings suggest that there may be differences in how neurophysiological resources are used to support behavioral performance during dual-tasking in PwMS compared to non-MS controls.

### 4.4 Motor-motor dual-task

In the motor-motor dual-task used in this study, participants performed a “toe-tapping” task under varied conditions of novelty and complexity while simultaneously performing a motor task involving maintenance of steady pinch grip force. Similar to the findings from the cognitive-motor dual-task, dual-task cost in pinch grip force performance resulted in few clear trends across participants. Likewise, CSP dual-task cost findings during the motor-motor dual-task followed a similar, but perhaps more discernible, trend as the observations made from the dual cognitive-motor task results. With the motor-motor dual-task, most PwMS demonstrated a clear lengthening of CSP duration under dual-task conditions. Non-MS controls also demonstrated a general lengthening of CSP duration under dual-task conditions, but the effects were lesser compared to those observed in PwMS. On average, CSP duration was lengthened in the dual motor-motor conditions relative to the single motor task by approximately 80% for PwMS and 6% for non-MS controls. These findings are partly in line with that of Corp et al. (9), in which CSP lengthening was observed in healthy adult participants aged 21–45 years old when performing a similar pinch grip task while pedaling on a stationary bike. In this study, the pinch grip task included an easy condition, where the pinch grip target force bar was presented visually as 15% ± 5% MVC, and a difficult condition with a bar presented as 15% ± 1% MVC. The authors found an increased CSP duration during dual-tasking; however, this effect was limited to the easy dual-tasking condition and was not observed during the more difficult condition. It was suggested that the difference in difficulty between the two conditions may not have been sufficient to elicit significant changes and, further, that the increased attentional demands during the more difficult condition may have caused a lesser upregulation of inhibition (9). It is challenging to draw comparisons with the current study, given population differences, although it is possible that regulation of corticospinal inhibition under varying dual-task demands differs between PwMS and non-MS controls. Speculation may include consideration of a general difference in GABA content throughout the CNS in PwMS which drives increased corticospinal inhibition during dual-tasking (13). Corp et al. (9) previously postulated that increasing corticospinal inhibition during dual-tasking might serve to minimize error that occurs as a result of distraction or divided attention. Potentially, PwMS are more susceptible to such error and require more corticospinal inhibition to counter such error and stabilize motor performance under dual-task conditions. Moreover, fatigue effects were likely to be induced in these trials given the requirement to maintain toe-tapping for approximately 200 s, including at a rapid pace. Such effects could potentially cause bilateral and interlimb changes in cortical excitability (65, 66) that may have also contributed to alterations in CSP duration in PwMS.

In consideration of protocol time constraints and other comparable literature, we did not collect toe-tapping data under single task conditions. Nevertheless, the toe-tapping component of the dual motor-motor task proved to be the only task in which differing levels of novelty and complexity had an apparent impact on performance. As shown in Figure 6C, the low novelty conditions produced lower variability among the majority of participants, whereas the high novelty conditions involving an external beat produced greater variability. The high novelty and high complexity condition generally resulted in the greatest variability among participants. Nevertheless, PwMS appeared to experience similar decrements in CSP dual-task cost across all conditions. While not specifically a dual-task paradigm, Goetschalckx et al. (67) examined how different metronome frequencies impacted rhythmic interlimb coordination in the lower extremities in PwMS, which the authors suggested requires both cognitive and motor control. It was determined that PwMS demonstrated greater variability in interlimb coordination than non-MS controls, with differences becoming more apparent in rhythms using more “deviant” frequencies. These results align to some extent with that of the current study, in which the more novel conditions caused increased variability in toe-tapping performance, albeit in both PwMS and non-MS controls (67). Together, these preliminary observations provide support for further investigation of dual motor-motor task behavior in PwMS.

### 4.5 Limitations

In addition to sample considerations addressed in Section 4.1 above, several other study limitations must be acknowledged. We characterized participants using the PDDS as a self-reported measure of subjective disability rather than the Expanded Disability Status Scale (EDSS) reported by a neurologist. Although not interchangeable with the EDSS, the PDDS is a simple, economical, efficient and valid assessment of disability in PwMS (68). The small sample size limited the capacity to explore whether disability status impacted results. Nevertheless, inclusion and exclusion criteria and the requirement of participants to be able to complete the tasks resulted in recruitment of individuals with similar disability profiles. Additionally, while reasonably well-matched for age and sex, the mean weight of non-MS controls was approximately 20 kg higher that PwMS in the study (Table 3). Of note, mean weight of the non-MS controls was increased by inclusion of two females who engage in athletics and regular strength training (Supplementary Table S1, NC-1 and NC-2), and for whom typical body mass index calculations may not be indicative of health status. Although group-based comparisons were not a focal point of this work and the impact of such factors on dual-tasking is not well-documented in prior literature, it is plausible that such baseline differences could influence dual-tasking behaviors and physiology.

Another limitation was the length of the study visit (~3 h) and potential of MS-related fatigue influencing results over the course of the session. The time required for collection of multiple task types and conditions along with TMS data was substantial, and ultimately precluded collection of some elements of data that could have been additive to results interpretation. Examples of other data that may have been of interest include additional cognitive tasks that emphasize other common MS-related cognitive deficits, such as executive function, and toe-tapping as a single task within the motor-motor paradigm. Instead, the protocol focused on considering different novelties and complexities of a cognitive task (23) that challenged working memory (43), a cognitive function considered to be highly susceptible to dual-task effects (42), and dual-task costs for the core task only under the motor-motor paradigm (9). Likewise, in some instances the protocol demands and extended timeline may have contributed to loss of data (e.g., portion of grip force data noted), although the majority of data was collected without issue. To mitigate any systematic influences of cumulative fatigue, the order of tasks and task conditions were randomized for each participant. Distributing the measurements across two sessions may have been an alternative approach to shorten visit durations. Yet, given that TMS stimulator outputs and the core motor task force target were referenced to measures (AMT, RMT, MVC) obtained at the beginning of the study protocol, breaking the study into multiple visits would have created the possibility for misrepresentation of changes in dual-task performance due to day-to-day variability. Considering fatigue, timing of medication dosage, and physical activity levels that vary day-to-day for individuals, completing the study in one session aided in ensuring that these factors did not alter the previously mentioned baseline variables necessary for determining the values utilized throughout the protocol. Moreover, large portions of the visit were dedicated to TMS hot-spotting, thresholding, task set-up, and instruction, which would all need to be re-conducted in a second session.

A further consideration is that there is no current gold standard protocol for evaluating dual-task performance in PwMS, nor with the incorporation of TMS. However, we followed recommendations posed in recent literature when developing the paradigms to measure performance in both tasks across different novelty and complexity conditions (23), and in a manner that allowed assessment of neurophysiological effects. For example, the specific cognitive task was chosen because prior studies have indicated that working memory impairment is common among PwMS and has shown to be exacerbated during dual-tasking (41, 42, 69, 70). Still, use of a unique, non-standardized cognitive assessment without evidence of concurrent or convergent validity or reliability limits interpretation. As a result, it must be considered that the cognitive performance results presented here may be task-specific and not directly comparable to other more established dual-task paradigms.

Finally, while TMS provides an avenue to study the neural basis of dual-tasking, it also presents limitations. TMS measures are inherently susceptible to variability from numerous sources, including factors related to the participant (e.g., alertness), the environment (e.g., distraction), and the user (e.g., coil position and orientation) (34, 71). Advanced modeling procedures to account for alterations in coil position and orientation are in development (72) but are not yet standard in the field. To minimize variability in the current work as per conventional procedures (34), we monitored participants closely during data collection, maintained a controlled data collection setting, and used standardized TMS procedures including application of neuronavigation software. Moreover, as used in this work, TMS provides a window into motor system activity only, without consideration of activity in other relevant CNS regions and structures, which may be better captured by other observational techniques such as functional magnetic resonance imaging or electroencephalography.

### 4.6 Future directions and clinical implications

To our knowledge, this is one of the first studies to explore the neurophysiological underpinnings of dual-tasking in PwMS. Additionally, few prior studies of PwMS have concurrently examined both cognitive-motor and motor-motor dual tasks that vary in levels of novelty and complexity. The size of the sample studied in the current work precluded the use of group-based inferential statistics, and accordingly constrained the conclusive interpretation of results. Nevertheless, this small-N study serves as an important starting point for more research in this topic area. Future work may attempt to replicate or confirm aspects of these findings (e.g., lengthened CSP with motor-motor dual-tasking in PwMS) with a larger sample size in order to determine statistical significance. Sample sizes employed in such work may be informed by the results reported here. Given the potential importance of understanding how dual-tasking is achieved in PwMS, future research should continue to explore mechanisms within the CNS (9, 60). Based on the preliminary evidence of increased corticospinal inhibition under dual-task conditions that emerged from the results, it appears that PwMS may be using different or more neural resources than those without MS, and this could have far-reaching impacts that we do not yet understand. For example, approaches to screen for physiological changes that precede behavioral declines, or treatments that normalize physiological changes to support motor and cognitive behavior might be developed. As such, current findings have potential to support generation of hypotheses for future study with different physiological measures and techniques. An improved understanding of neurophysiological differences could aid in developing science-based rehabilitation strategies that target cognitive-motor or motor-motor interference impairments experienced by PwMS. Development of such strategies will also require enhanced understanding of the apparent disconnect between reports from PwMS of impaired dual-tasking and lack of evidence for impaired performance beyond that measured in non-MS controls, such as that observed in the current work and elsewhere (22). Furthermore, findings from this study, which incorporated a variety of single and dual-task conditions, may be drawn on in future work focused on development of streamlined, standardized protocols for administering dual-tasks within PwMS.

## Data Availability

The raw data supporting the conclusions of this article will be made available by the authors, without undue reservation.
