# Supplementary material for: Considering neurophysiological mechanisms of dual-tasking in people with multiple sclerosis: an exploratory, cross-sectional small-N study
Source: Front Neurol. 2026 Jan 30;17:1648874. doi: 10.3389/fneur.2026.1648874 (PMC12900680; doi:10.3389/fneur.2026.1648874)
Supplement: Supplementary file 1 [file Table_1.docx]

**Table S.1.** Characteristics of individual participants.

|  | **PwMS-1** | **PwMS-2** | **PwMS-3** | **PwMS-4** | **PwMS-5** | **PwMS-6** | **NC-1** | **NC-2** | **NC-3** |  |
| --- | --- | --- | --- | --- | --- | --- | --- | --- | --- | --- |
| Age (years) | 29 | 34 | 34 | 46 | 61 | 67 | 27 | 40 | 60 |  |
| Sex | F | F | F | F | M | M | F | F | M |  |
| Height (m) | 1.80 | 1.65 | 1.65 | 1.65 | 1.75 | 2.01 | 1.74 | 1.6 | 1.71 |  |
| Weight (kg) | 82 | 79.38 | 94.35 | 79.38 | 117 | 90.72 | 106.59 | 90.7 | 136.08 |  |
| MS Phenotype | RRMS | RRMS | RRMS | RRMS | PPMS | PPMS | - | - | - |  |
| Disease Duration (years) | 2 | 6 | 4 | 19 | 4 | 3 | - | - | - |  |
| Taking DMTs (Y/N) | Y | Y | Y | Y | Y | N | - | - | - |  |
| PDDS | 1 | 2 | 4 | 6 | 5 | 6 | - | - | - |  |
| SymptoMScreen | 14 | 17 | 14 | 28 | 24 | 30 | - | - | - |  |
| MSIS-29 | 62 | 51 | 56 | 121 | 81 | 61 | - | - | - |  |
| DTQ-Expanded | 2.40 | 2.60 | 1.53 | 3.53 | 2.20 | 3.13 | 2.40 | 2.00 | 2.93 |  |

*Note.* F, female. M, male. RRMS, Relapse Remitting Multiple Sclerosis. PPMS, Primary Progressive Multiple Sclerosis. DMT, disease modifying therapies. PDDS, Patient-determined Disease Steps. MSIS-29, Multiple Sclerosis Impact Scale. DTQ-Expanded, Dual-task Questionnaire-Expanded. PDDS ranges from 0-8, with a higher score indicating greater perceived disability. The SymptoMScreen is scored out of 72, with higher scores indicating greater impairment. MSIS-29 is scored out of 100, with higher scores indicating greater levels of disability. DTQ-Expanded is scored on a Likert scale (range 0.0-4.0) and averaged across 15 questions, with a higher score indicating greater perceived difficulties with dual-tasks.
